# Synthesis and Biological Evaluation of Novel Curcuminoid Derivatives

**DOI:** 10.3390/molecules191016349

**Published:** 2014-10-13

**Authors:** Ya-Kun Cao, Hui-Jing Li, Zhi-Fang Song, Yang Li, Qi-Yong Huai

**Affiliations:** 1 Department of Applied Chemistry, Marine College, Shandong University, Weihai 264209, China; E-Mails: yakun_cao@163.com (Y.-K.C.); zhifangdl@163.com (Z.-F.S.); LYchemlh@hotmail.com (Y.L.); 2 Department of Environmental Engineering, School of Marine Science and Technology, Harbin Institute of Technology, Weihai 264209, China; E-Mail: lihuijing@iccas.ac.cn

**Keywords:** curcuminoids, antibacterial, antioxidant, antiproliferative

## Abstract

Curcuminoids have been reported to possess multiple bioactivities, such as antioxidant, anticancer and anti-inflammatory properties. Three novel series of curcuminoid derivatives (**11a**–**h**, **15a**–**h** and **19a**–**d**) with enhanced bioactivity have been synthesized. Among the synthesized compounds, **11b** exhibited the most significant activity with an MIC of 0.5 µM /mL against selected medically important Gram-positive cocci (*S. aureus* and *S. viridans*) and Gram-negative bacilli (*E. coli* and *E. cloacae*). The derivatives exhibited remarkable results in an antioxidant test with an IC_50_ 2.4- to 9.3-folder smaller than curcuminoids. With respect to antiproliferative activity against Hep-G2, LX-2, SMMC7221 and MDA-MB-231, the derivatives exhibited an effect stronger than curcuminoids with an IC_50_ ranging from 0.18 to 4.25 µM.

## 1. Introduction

**Figure 1:**
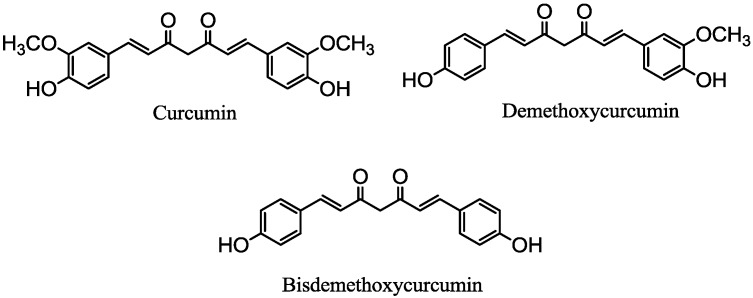
Curcumin, demethoxycurcumin and bisdemethoxycurcumin.

Natural compounds derived from plants, animals and microorganisms proved to be an excellent source for therapeutic agents. Phytochemicals once served humankind as the source of new drug development, and today, these natural products or their derivatives still represent preventive and therapeutic agents against a wide range of human diseases [1,2]. Turmeric and ginger are spices that belong to the Zingiberaceae family. Ginger, a common ingredient for various foods and beverages, is also employed in medicine for the treatment of various ailments, like headaches, nausea, rheumatism and colds [3]. Curcuminoids, *viz*., curcumin (CCM), demethoxycurcumin (DCM) and bisdemethoxycurcumin (BCM), are the yellow pigments of turmeric [4]. (Figure 1) Recent studies have shown that curcuminoids possess a wide range of biological activities, including antioxidant [5,6], anti-inflammatory [7,8], anticancer [9,10] and anti-angiogenesis [11,12] properties, as well as medicinal applications, including their use in HIV therapies [13] and Alzheimer’s disease [14]. The non-toxic food origin and wide range of pharmaceutical properties of curcuminoids make them promising candidate molecules for medicine. Efforts have been made to synthesize new curcuminoid derivatives with stronger biological activities [15,16,17].

In addition to curcuminoids, some other natural products, such as methionine [18,19], selenomethionine [19,20,21,22], caffeic acid [23,24,25], ferulic acid [26,27,28] and 18β-glycyrrhetinic acid [29,30,31], have drawn our attention because of various pharmaceutical properties. Hence, we selected those active ingredients as starting materials to design and synthesize three serials of curcuminoid derivatives. The antioxidant ability, antibacterial activity, as well as cytotoxic potency of the compounds against human tumor cells were evaluated.

## 2. Results and Discussion

### 2.1. Chemistry

**Scheme 1:**
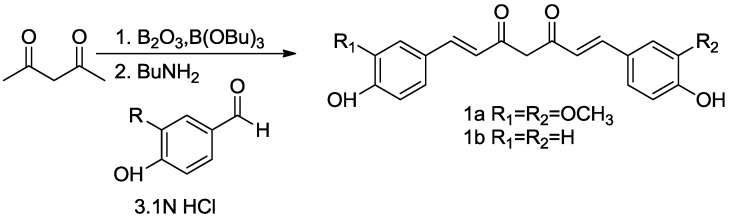
Syntheses of curcumin (**1a**) and bisdemethoxycurcumin (**1b**).

Symmetrical curcuminoids, CCM and BCM were synthesized according to the procedure previously reported (Scheme 1) [32]. Compounds **1a** and **1b** were obtained in an excellent yield. NMR and other analytical data support the curcuminoids’ purity of >95% after recrystallization in methanol.

**Scheme 2:**
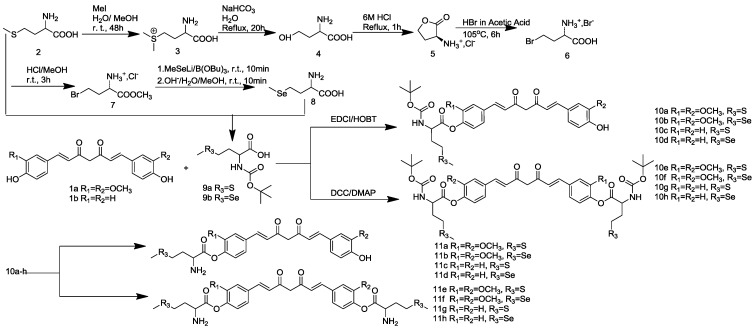
Synthesis of selenomethionine-substituted curcuminoids and methionine-substituted curcuminoids.

The synthesis of curcuminoid derivatives, *viz*., **11a**–**h**, **15a**–**h** and **19a**–**d**, were reported. In the first series, selenomethionine (8) was prepared from methionine (2) through seven steps according to the procedure previously reported (Scheme 2) [33]. The synthesis of **10a**–**h** was carried out in dry CHCl_3_ in the presence of dehydrating agents, 1-Ethyl-3-(3-dimethyllaminopropyl)carbodiimide hydrochloride/1-Hydroxybenzotriazole (EDCI/HOBT) or Dicyclohexylcarbodiimide/4-dimethylaminopyridine (DCC/DMAP), as catalysts. Subsequently, deprotection of amino group were completed with HCl in dry ethyl acetate (EA) at room temperature for 2–4 h to get methionine-substituted and selenomethionine-substituted curcuminoids (**11a**–**h**).

**Scheme 3:**
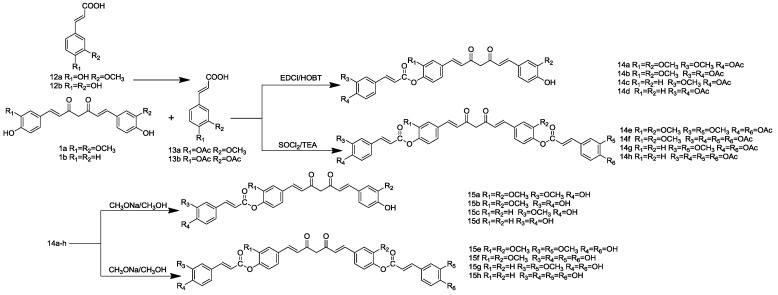
Synthesis of curcuminoid-caffeic acid and curcuminoid-ferulic acid conjugates.

**Scheme 4:**
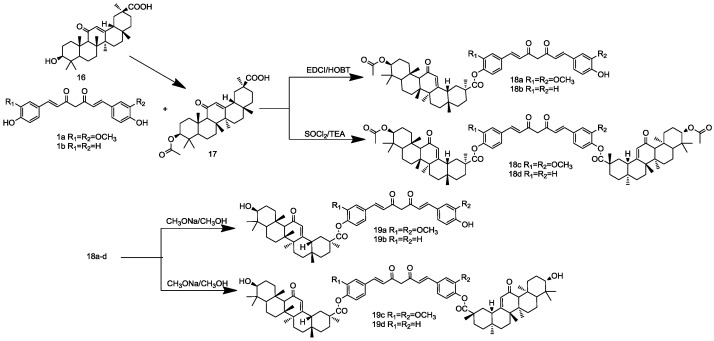
Synthesis of curcuminoid-glycyrrhetinic acid conjugates.

The synthesis of the second series depicted in Scheme 3, **13a** or **13b**, was reacted with curcuminoids in the presence of EDCI/HOBT to obtain **14a**–**d** in 45%–65% yield. Treatment of **13a** or **13b** with SOCl_2_ was followed by the addition of curcuminoids and Triethylamine (TEA) as the acid-binding agent, using the esterification procedure furnished compounds **14e**–**h**. Acetylated compounds **14a**–**h** resulted upon hydrolysis with CH_3_ONa, resulting in their respective phenolic compounds, **15a**–**h**. As shown in Scheme 4, we designed the synthesis of curcuminoid-glycyrrhetinic acid conjugates (**19a**–**d**) using similar methods. All of the newly synthesized bioconjugates were characterized by melting point, elemental analysis, ESI-MS, ^1^H-NMR and ^13^C-NMR.

### 2.2. Pharmacological Activities

#### 2.2.1. Antioxidant Activity

As one of their well-known mechanisms, antioxidants can inhibit lipid oxidation by scavenging free radicals. Compared to other methods of measuring antioxidant activity, the DPPH radical method is quite simple and rapid for screening specific compounds, as the DPPH radical is commercially available and does not need to be generated prior to use. Thus, the antioxidant activity of all of the synthesized new curcuminoid derivatives (**11a**–**h**, **15a**–**h** and **19a**–**d**) was assessed for their radical scavenging ability using the stable DPPH radical method. The assay was conducted at six different concentrations of antioxidants (2.5 µM, 5 µM, 10 µM, 20 µM, 40 µM and 80 µM) in a polar, homogeneous medium. The antioxidant potency (IC_50_) is presented in µM concentrations of the synthesized curcuminoid derivatives, as shown in Table 1. Besides, we tracked the change of absorbance of the DPPH solution over time in the presence of curcuminoid derivatives at a concentration of 20 µM and, based on these data, determined the decay rates of the DPPH radicals (Table 1).

**Table 1:** *In vitro* DPPH free radical scavenging activity (FRSA) of the target compounds. CCM, curcumin; BCM, bisdemethoxycurcumin.

Compound	DPPH FRSA%						IC_50_ (µM)	d[radical]/dt(µM·min^−1^)
	2.5 µM	5 µM	10 µM	20 µM	40 µM	80 µM		
**11a**	4.2	8.3	16.7	33.3	66.7	93.3	30	9.49
**11b**	11.4	22.7	45.5	90.9	95.5	96.6	11	18.58
**11c**	3.5	6.9	13.9	27.8	55.6	92.4	36	8.92
**11d**	6.3	11.9	23.8	47.6	92.2	95.5	21	13.14
**11e**	3.3	6.5	13.2	26.3	52.6	92.2	38	8.57
**11f**	5.2	10.3	19.8	39.8	81.5	94.8	25	12.44
**11g**	2.9	6.1	11.5	23.8	47.2	91.2	42	8.61
**11h**	4.5	8.8	17.6	35.5	71.2	93.9	28	11.05
**15a**	5.2	10.2	20.6	41.7	82.9	93.7	24	12.62
**15b**	3.4	6.6	13.5	27.3	54.9	93.1	37	8.83
**15c**	4.3	8.5	17.2	34.7	69.2	94.2	29	13.22
**15d**	4.6	9.2	18.5	37.3	74.1	93.9	27	11.55
**15e**	4.4	8.9	17.5	34.5	68.7	93.5	29	10.88
**15f**	3.1	6.3	11.9	23.5	47.6	92.2	42	7.25
**15g**	6.3	12.5	25.3	50.5	92.6	95.2	20	14.12
**15h**	3.4	6.6	13.9	27.3	54.1	92.8	37	10.23
**19a**	3.3	6.5	13.4	26.5	52.6	92.2	38	10.92
**19b**	2.6	4.9	10.3	20.8	40.8	80.7	49	5.89
**19c**	5.2	10.4	20.8	41.7	83.3	93.4	24	13.02
**19d**	3.6	7.1	14.4	28.6	57.1	92.3	35	9.88
**CCM**	1.2	2.5	4.9	9.7	19.5	38.8	103	1.34
**BCM**	1.2	2.3	4.1	8.5	16.3	32.1	123	0.56

The radical-scavenging assay by the DPPH method clearly indicated that all curcuminoid derivatives have lower IC_50_ values than curcuminoids. For instance, methionine-substituted curcumin exhibited 2.5- to 3.4-fold lower IC_50_ values than did curcumin as the standard compound, which could be attributed to the sulfur moiety present in it, acting as a good radical-scavenging agent. However, selenomethionine-substituted curcuminoids showed 1.5- to 2.7-fold lower IC_50_ values than did methionine-substituted curcuminoids, which revealed the important role of selenium in scavenging radicals.

The results also showed that attachment of a caffeic acid and a ferulic acid moiety to the curcuminoids significantly enhanced the antioxidant activity. For instance, curcumin-di-caffeic acid (**15f**) showed a potent DPPH free radical scavenging ability with IC_50_ of 29 µM, while the IC_50_ of curcumin as the standard compound is 103 µM. In addition, curcuminoids with a caffeic acid moiety showed a higher DPPH radical scavenging ability than those with ferulic acid, which revealed the great influence of free hydroxyl groups on the DPPH radical scavenging activity of curcuminoid derivatives. For instance, curcumin-mono-caffeic acid (**15b**) exhibited better antioxidant activity with an IC_50_ of 24 µM, while the corresponding curcumin-mono-ferulic acid (**15a**) showed an IC_50_ of 37 µM.

With respect to the third series of curcuminoid derivatives, the best radical scavenger is curcumin-di-glycyrrhetinic acid (**19c**), whose scavenging ability is 4.3-folder higher than curcumin as the standard compound. The curcuminoid-glycyrrhetinic conjugates also showed more efficiency than curcuminoids. As the results revealed, the curcumin-di-glycyrrhetinic acid exhibited a 9.7-folder higher scavenging radical rate than curcumin did.

#### 2.2.2. Antibacterial Activity

All curcuminoid derivatives were tested for their antimicrobial potential against Gram-positive cocci (*S. aureus* and *S. viridans*) and Gram-negative bacilli (*E. coli* and *E. cloacae*). The lowest concentration of curcuminoid derivatives in µmol/mL that prevented *in vitro* growth of microorganism has been represented as being correlated with the zone of inhibition and MIC (minimum inhibitory concentration), as shown in Table 2.

**Table 2:** Zone of inhibition ^a^ and MIC ^b^ correlation diagram of curcuminoid derivatives against bacterial strains.

Compound	Name of Bacteria							
	Gram-positive				Gram-negative			
	*S. aureus*		*S. viridans*		*E. coli*		*E. cloacae*	
	Zone of Inhibition	MIC	Zone of Inhibition	MIC	Zone of Inhibition	MIC	Zone of Inhibition	MIC
**11a**	19, 16, 14	2.0	19, 17, 14	2.0	19, 17, 15	2.0	18, 16, 14	4.0
**11b**	26, 20, 18	0.5	24, 20, 18	0.5	25, 20, 17	0.5	23, 20, 18	0.5
**11c**	19, 17, 15	2.3	18, 16, 14	2.3	19, 16, 14	4.6	19, 16, 14	4.6
**11d**	24, 17, 14	1.0	22, 18, 15	1.0	22, 17, 15	1.0	24, 19, 16	1.5
**11e**	18, 15, 13	3.2	18, 14, 10	2.2	18, 15, 13	3.2	18, 14, 13	3.2
**11f**	21, 18, 16	0.7	21, 18, 16	0.7	20, 17, 15	0.3	21, 18, 15	0.7
**11g**	18, 16, 14	3.7	22, 19, 17	2.4	18, 16, 13	3.7	20, 18, 16	2.4
**11h**	20, 17, 15	1.6	23, 20, 18	1.6	20, 15, 13	1.6	22, 19, 17	1.6
**15a**	19, 16, 14	3.8	19, 17, 14	3.8	19, 17, 14	2.4	19, 16, 15	1.9
**15b**	22, 20, 18	3.7	21, 19, 16	3.7	22, 20, 18	2.3	22, 20, 18	1.8
**15c**	18, 15, 13	4.3	19, 15, 13	4.3	18, 16, 11	2.7	18, 15, 12	2.1
**15d**	20, 18, –	4.1	20, 18, –	4.1	20, 18, –	2.6	21, 18, 17	2.1
**15e**	18, 15, 13	2.7	18, 15, –	2.7	16, 13, 10	1.7	19, 17, 13	1.3
**15f**	21, 17, 16	2.8	23, 17, 16	2.8	21, 17, 16	1.7	21, 18, 16	1.4
**15g**	19, 17, 14	3.2	19, 16, 14	3.2	19, 16, 14	2.0	19, 16, 14	1.6
**15h**	21, 19, 18	3.0	26, 20, 18	3.0	26, 20, 18	1.9	26, 20, 18	1.5
**19a**	19, 17, 15	2.4	20, 18, 16	2.4	20, 18, 17	1.5	19, 17, 16	1.2
**19b**	21, 19, 17	2.6	21, 18, 16	2.6	21, 18, 17	1.6	20, 18, –	1.3
**19c**	25, 20, 18	1.6	24, 21, 19	1.6	24, 22, 20	1.0	23, 21, 19	0.8
**19d**	22, 20, 18	1.7	22, 20, 18	1.7	22, 19, 17	1.0	21, 19, 17	0.8
**CCM**	10, 8, 6	21.7	11, 9, –	21.7	10, 8, –	27.2	12, 8, –	27.2
**BCM**	8, 5, –	32.5	8, 6, –	32.5	8, 5, –	32.5	9, 6, –	32.5
**Ampicillin**	21, 18, 15	2.5	20, 17, 15	2.5	20, 17,14	3.2	20, 18, 15	3.2

– Resistant; ^a^ the zone of inhibition was measured in mm at concentrations of 20, 10 and 5 µM/mL; ^b^ MIC (minimum inhibitory concentration) values were measured in µM/mL.

All curcuminoid derivatives exhibited better antibacterial activity than curcuminoids. The most encouraging results were obtained in the case of curcumin-mono-selenomethionine (**11b**) having an MIC of 0.5 µM/mL against *S. aureus*, while ampicillin, the best marketed antibiotic, shows an MIC of 2.5 µM/mL, showing that (**11b**) is five times more effective than the ampicillin at similar concentrations. The zones of inhibition of **11b** were 26 mm, 20 mm and 18 mm, while curcumin had zones of 10 mm, 8 mm and 6 mm. The monoester **11b** has shown highly satisfactory results as an antibacterial agent, which may be due to the selenium and free hydroxyls. Curcuminoid-caffeic acid and curcuminoid-ferulic acid conjugates showed an MIC ranging from 2.8 to 4.3 µM/mL. With respect to the third series of curcuminoid derivatives, the best antibacterial agents are **19c** and **19d**, with an MIC ranging between 1.6 µM/mL and 1.7 µM/mL, 1.5- to 1.6-fold lower than did ampicillin.

What is more, the second and third series of curcuminoid derivatives showed better antibacterial activity against *E. coli* than ampicillin without exception. Curcuminoid-caffeic acid and curcuminoid-ferulic acid conjugates showed an MIC ranging between 1.7 µM/mL and 2.7 µM/mL, 1.2- to 1.9-folder lower than ampicillin did. Curcumin-glycyrrhetinic acid conjugates showed an MIC ranging between 0.8 µM/mL and 1.3 µM/mL, 2.5- to 4.0-folder lower than ampicillin did.

The results of an antibacterial test against *E. cloacae* were also encouraging. The antibacterial activity of the bioconjugates was 8.6–49.4-times higher than that of curcuminoids.

#### 2.2.3. Antiproliferative Activity

The inhibitory effects of curcuminoid derivatives on the growth of three lines of cultured human hepatoma cells (Hep-G2, LX-2 and SMMC-7221) and one line of human breast cancer cells (MDA-MB-231) were determined using the MTT assay. For each experiment, CCM and BCM were used as positive controls. The cytotoxic effects of curcuminoid derivatives were confirmed at concentrations of 1 µM, 5 µM, 10 µM and 20 µM. Inhibitory activities (IC_50_) were presented in µM/L concentrations of the synthesized curcuminoid derivatives, as shown in Table 3.

All curcuminoid derivatives showed remarkably stronger cancer growth inhibitory effects in Hep-G2, LX-2, SMMC-7221 and MDA-MB-231 cells than did curcuminoids, as determined by the MTT assay. The activity data suggests that the derivatives having methionine exhibited good anticancer activity, and replacement of the selenium atom with the sulfur atom showed more remarkable anticancer activity, which was effective against all cell lines. Compound **11b** exhibited an IC_50_ of 0.18–0.63 µM in Hep-G2, LX-2, SMMC-7221 and MDA-MB-231 cells, while the IC_50_ of **11a** was 4.9- to 9.8-fold higher (1.41–2.94 µM). We also determined that the monoesters demonstrated improved anticancer properties compared to their corresponding diesters. The ester linkages to selenomethionine and methionine could be hydrolyzed by esterase enzymes, releasing parent curcuminoids at the target site. Thus, monoesters having one free phenolic hydroxyl and one ester linkage to selenomethionine have great advantages and hold promise as prodrugs.

In the second series of curcuminoid derivatives, curcuminoid-caffeic acid conjugates showed a stronger inhibition effect on the growth of tumor cells than curcuminoid-ferulic acid conjugates. For instance, Compound **15a** exhibited an IC_50_ of 2.75–3.96 μM in Hep-G2, LX-2, SMMC-7221 and MDA-MB-231 cells, while the IC_50_ of **15b** was between 1.11 and 2.16 μM.

**Table 3:** Inhibitory effects of curcuminoid derivatives on the growth of Hep-G2, LX-2, SMMC7221 and MDA-MB-231.

Compound	IC_50_ (µM)			
	Hep-G2	LX-2	SMMC-7721	MDA-MB-231
**CCM**	24.95	13.97	12.57	11.45
**BCM**	32.65	16.35	17.11	14.10
**11a**	2.54	2.92	2.05	2.64
**11b**	0.31	0.62	0.18	0.52
**11c**	3.01	3.22	2.35	1.84
**11d**	0.75	0.98	0.55	0.85
**11e**	3.54	3.55	3.41	3.59
**11f**	0.95	0.78	1.35	1.22
**11g**	4.01	4.39	4.17	4.62
**11h**	0.96	1.42	1.21	1.82
**15a**	3.75	3.96	2.75	3.23
**15b**	1.11	1.28	2.16	1.72
**15c**	4.25	3.36	3.32	2.75
**15d**	3.11	1.28	1.16	2.05
**15e**	5.15	4.24	3.23	3.05
**15f**	2.16	3.38	2.98	2.80
**15g**	3.75	3.94	2.75	3.23
**15h**	3.11	3.28	2.16	2.72
**19a**	4.25	3.36	3.32	2.75
**19b**	3.16	2.25	2.28	2.15
**19c**	0.75	0.94	1.03	1.05
**19d**	1.52	1.48	1.65	1.78

IC_50_, the compound concentration required to inhibit cell proliferation by 50%. Hep-G2, LX-2, SMMC-7221 and MDA-MB-231 were seeded at a density of 2 × 10^3^ Cells/well. The cells were then treated with various concentrations (1 µM, 5 µM, 10 µM and 20 µM) of different curcuminoid derivatives for 72 h. The effects of the different compounds on the growth of Hep-G2, LX-2, SMMC-7221 and MDA-MB-231 cells were determined by the MTT assay.

Among the curcuminoid-glycyrrhetinic acid conjugates, **19c** showed the best antiproliferative activity with an IC_50_ of 0.75–1.05 µM. In the first series, we found that the monoesters exhibited better anti-tumor activity than their corresponding diesters, but the third series is just the opposite. The IC_50_ of curcumin-mono-glycyrrhetinic acid (**19a**) ranged between 2.75 to 4.25 µM, while the IC_50_ of curcumin-di-glycyrrhetinic acid (**19c**) was between 0.75 µM and 1.05 µM.

### 2.3. Structure-Activity Relationship

Curcumin has two phenyl rings and substitutions at the 3 and 4 positions with methoxy and hydroxyl groups, respectively. It has been reported to undergo extensive *in vitro* and *in vivo* phase I and phase II metabolism through oxidation, reduction, glucuronidation and sulfation. The glucuronidation and sulfation occur on the 4-OH groups present on both phenyl rings of curcumin. The protection of the 4-OH groups improves its stability.

In the first series of curcuminoid derivatives, the assessment of their antioxidant, antimicrobial and anticancer activities suggested that derivatives are relatively more active than curcuminoids, which may be due to their increased solubility, slow metabolism and better cellular uptake. Shiv K. Dubey *et al.* [34] enhanced the biological activities of curcumin by introducing glycine, glutamic acid and valine on phenyl rings. They also found that monoesters of curcumin have even better antimicrobial activity than their corresponding diesters, emphasizing the role of the free phenolic group. That findings are consistent with our results. What is more, the CCM derivatives exhibited better bioactivities than the corresponding BCM derivatives, which revealed the importance of methoxy groups in the skeleton for biological activity.

Wisut *et al.* [35] synthesized a series of succinate prodrugs by esterification of curcuminoids with a methyl or ethyl ester of succinyl chloride. These compounds showed better anticancer activity against Caco-2 cells with IC_50_ values in the 1.8–9.6 µM range, Compared with succinylation, the conjugation of caffeic acid and ferulic acid is in favor of the formation of hydrogen bond formation and the extension of the conjugate structure. Zhang’s [36] research found that these factors greatly impact the bioactivity of curcuminoid derivatives. In addition, the curcuminoids-caffeic acid conjugates showed better activities than curcuminoids-ferulic acid conjugates, which may be attributed to different numbers of hydroxyl groups in two substituents.

In the third series, 18β-glycyrrhetinic acid was used as the starting material to prepare prodrugs with novel structures. To alter the lipophilicity of the molecule and to improve the bioavailability, the carboxylic acid at position C-30 was transformed into esters by reacting with curcuminoids. The synthesized derivatives retained the active groups of 18β-glycyrrhetinic acid, the C-3 hydroxyl group, which proved to be essential for good biological activities. In addition, diesters with a two glycyrrhetinic acid moiety showed better biological activities than monoesters, and the results agree with Fuchs’ findings [37].

Thus, we attempt to summarize the key issues:

1. The absence of the methoxy group in curcuminoid derivatives leads to a decrease in the biological activity.
2. The difference in the biological activity between monoesters and diesters depends on the substituent on the phenyl rings of curcuminoids.
3. To some extent, but not as hard absolutes, the more hydroxyl groups the substituent contains, the better biological activity the derivatives will have. In other word, the hydroxyl groups incorporated are helpful for the improvement of biological activity.

## 3. Experimental Section

### 3.1. Chemicals and Reagents

All reagents and solvents were purchased from Aldrich Chemical Co. (Beijing, China) and used without further purification. Se-Met was synthesized in-house. Melting points were determined on a Fisher-Johns melting apparatus (Weihai, China). Electrospray ionization mass spectral (ESI-MS) data were obtained on a Bruker Esquire HCT spectrometer (Waters, New York, NY, USA). The ^1^H-NMR spectral data were recorded on a 400-MHz NMR spectrometer (Fällanden, Switzerland).

Human hepatoma cells (Hep-G2, LX-2 and SMMC-7221) and human breast cancer cells (MDA-MB-231) were obtained from Expression Systems (Beijing, China). Cultured cells were grown at 37 °C in a humidified atmosphere of 5% CO_2_ and were passaged twice a week.

### 3.2. General Method for the Synthesis of CCM (**1a**) and BCM (**1b**)

Acetylacetone (10 mmol) was added to a solution of boric anhydride (5.0 mmol) in ethyl acetate (30 mL), followed by the addition of vanillin (20 mmol) or 4-hydroxybenzaldehyde (20 mmol) and tributyl borate (40 mmol). The reaction mixture was stirred at 50 °C for 10 min. Subsequently, *n*-butylamine (5.0 mmol) in ethyl acetate (5 mL) was added dropwise over 15 min at 50 °C and additionally stirred for 4 hours at 80 °C. Hydrochloric acid (1 N, 30 mL) was added, and the mixture was stirred for another 40 min. The organic layers were washed with water, dried with Na_2_SO_4_. The solvent was evaporated, and the residue was recrystallized from methanol to give the corresponding curcuminoids (**1a** and **1b**) as yellow solids.

CCM (**1a**). Yield 85%. m.p. 185–186 °C. ^1^H-NMR (CDCl_3_): δ 7.59 (d, *J =* 15.7 Hz, 2H), 7.13 (dd, *J =* 8.1 and 1.8 Hz, 1H), 7.05 (d, *J* = 1.8 Hz, 2H), 6.94 (d, *J* = 8.1 Hz, 2H), 6.48 (d, *J* = 15.8 Hz, 2H), 5.85 (s, 1H), 3.95 (s, 6H). ESI-MS (*m/z*): (M−H)^−^ = 367.03. Anal. Calcd. for C_21_H_20_O_6_: C, 68.47; H, 5.47; O, 26.06%. Found: C, 68.40; H, 5.41; O, 26.12%.

BCM (**1b**). Yield 80%. m.p. 233–234 °C. ^1^H-NMR (CDCl_3_): δ 7.53 (d, *J* = 16.2 Hz, 2H).7.55 (d, *J =* 8.6 Hz, 4H), 6.85 (d, *J* = 8.6 Hz, 4H), 6.65 (d, *J* = 15.9 Hz, 2H), 6.09 (s, 1H). ESI-MS (*m/z*): (M−H)^−^ = 307.03. Anal. Calcd. for C_21_H_20_O_6_: C, 74.01; H, 5.23; O, 20.76%. Found: C, 74.06; H, 5.21; O, 20.82%.

### 3.3. Procedure of the Preparation of Selenomethionine-Substituted Curcuminoids and Methionine-Substituted Curcuminoids

According to the procedure previously reported [33], selenomethionine (8) was prepared. Methionine (2) was used as the starting material in a three-pot, seven-step procedure to obtain selenomethionine in 47% yield. In a 100-mL round-bottomed flask, selenomethionine or methionine was dissolved in water and NaHCO_3_ (3 eq.) was added. A solution of (BOC)_2_O (1.5 eq.) in dioxane was added to this mixture. The reaction mixture was stirred at 25 °C overnight. The reaction mixture was washed with ethyl acetate. The resulting aqueous layer was acidified to pH 2 with concentrated hydrochloric acid and extracted with ethyl acetate. The combined organic extracts were dried over Na_2_SO_4_, filtered and concentrated *in vacuo* to give **9a** or **9b** as colorless gums.

#### 3.3.1. General Procedure for the Synthesis of **10a**–**d**

To a solution of **9a** or **9b** (10 mmol) in chloroform (50 mL) was added EDCI (12 mmol), HOBT (12 mmol) and DIEA (1 mL). After stirring at 0 °C for 1 h, a solution of CCM (10 mmol) or BCM (10 mmol) in chloroform (40 mL) was added dropwise to the reaction mixture, which was stirred overnight at room temperature. After completion of the reaction, as indicated by TLC, the mixture was washed with hydrochloric acid (1 M) and brine. The organic layer was dried over anhydrous Na_2_SO_4_ and concentrated *in vacuo*. Purification by column chromatography over polyamide gel (1:10, CH_3_OH/CCl_4_) gave **10a**–**d** as yellow solids.

*Curcumin-mono-N-(tert-butoxycarbonyl)-methionine* (**10a**). Yield 48%. m.p. 165–166 °C. ^1^H-NMR (400 MHz, CDCl_3_): δ 7.63 (d, *J* = 5.6 Hz, 1H), 7.59 (d, *J* = 5.6 Hz, 1H), 7.21–7.07 (m, 4H), 7.06 (d, *J* = 1.8 Hz, 1H), 6.94 (d, *J* = 8.2 Hz, 1H), 6.55 (d, *J* = 15.8 Hz, 1H), 6.49 (d, *J* = 15.8 Hz, 1H), 5.83 (s, 1H), 5.21 (d, *J =* 8.0 Hz, 1H), 4.71 (s, 1H), 3.95 (s, 3H), 3.87 (s, 3H), 2.69 (t, *J =* 7.8 Hz, 2H), 2.34 (m, 2H), 2.15 (s, 3H), 1.47 (s, 9H). ^13^C-NMR (CDCl_3_): δ 198.9, 173.4, 155.2, 151.4, 149.5, 147.9, 142.8, 141.1, 130.6, 127.6, 122.2, 121.9, 116.8, 112.5, 110.9, 79.5, 56.5, 55.8, 51.9, 30.9, 29.7, 28.4. ESI-MS (*m/z*): (M−H)^−^ = 598.31. Anal. Calcd. for C_31_H_37_NO_9_S: C, 62.09; H, 6.22; N, 2.34%. Found: C, 62.12; H, 6.24; N, 2.38%.

*Curcumin-mono-N-(tert-butoxycarbonyl)-selenomethionine* (**10b**). Yield 52%. m.p. 172–173 °C. ^1^H-NMR (400 MHz, CDCl_3_): δ 7.63 (d, *J =* 6.0 Hz, 1H), 7.59 (d, *J =* 6.0 Hz, 1H), 7.22–7.07 (m, 4H), 7.06 (d, *J =* 1.6 Hz, 1H), 6.94 (d, *J =* 8.2 Hz, 1H), 6.56 (d, *J =* 15.8 Hz, 1H), 6.50 (d, *J =* 15.8 Hz, 1H), 5.83 (s, 1H), 5.20 (d, *J =* 8.1 Hz, 1H), 4.71 (d, *J =* 5.0 Hz, 1H), 3.95 (s, 3H), 3.87 (s, 3H), 2.75–2.67 (m, 2H), 2.41–2.21 (m, 2H), 2.05 (s, 3H), 1.47 (s, 9H). ^13^C-NMR (CDCl_3_): δ 198.5, 172.4, 155.9, 152.3, 149.5, 148.6, 142.6, 142.5, 133.8, 131.4, 125.6, 124.3, 122.9, 121.9, 116.8, 111.9, 110.9, 56.1, 55.8, 51.9, 28.4, 25.1. ESI-MS (*m/z*): (M−H)^−^ = 646.08. Anal. Calcd. for C_31_H_37_NO_9_Se: C, 57.58; H, 5.77; N, 2.17%. Found: C, 57.50; H, 5.71; N, 2.12%.

*Bisdesmethoxycurcumin-mono-N-(tert-butoxycarbonyl)-methionine* (**10c**). Yield 55%, m.p. 183–184 °C. ^1^H-NMR (400 MHz, CDCl_3_): δ 7.61 (d, *J =* 12.9 Hz, 2H), 7.54 (d, *J =* 8.3 Hz, 2H), 7.43 (d, *J =* 8.1 Hz, 2H), 7.12 (d, *J =* 8.5 Hz, 2H), 6.85 (d, *J =* 7.8 Hz, 2H), 6.56 (d, *J =* 15.8 Hz, 1H), 6.48 (d, *J =* 15.8 Hz, 1H), 5.80 (s, 1H), 5.24 (d, *J =* 7.8 Hz, 1H), 4.67 (s, 1H), 2.66 (t, *J =* 8.1 Hz, 2H),2.25 (m, 2H) 2.14 (s, 3H), 1.48 (s, 9H). ^13^C-NMR (CDCl_3_): δ 198.5, 171.4, 158.8, 153.9, 150.2, 134.8, 131.9, 130.8, 130.2, 129.6, 121.6, 116.8, 79.2, 56.8, 51.9, 30.6, 28.6, 15.6. ESI-MS (*m/z*): (M−H)^−^ = 538.33. Anal. Calcd. for C_29_H_33_NO_7_S: C, 64.54; H, 6.16; N, 2.60%. Found: C, 64.50; H, 6.11; N, 2.65%.

*Bisdemethoxycurcumin-mono-N-(tert-butoxycarbonyl)-selenomethionine* (**10d**). Yield 60%. m.p. 192–193 °C. ^1^H-NMR (400 MHz, CDCl_3_): δ 7.61 (d, *J =* 14.9 Hz, 2H), 7.54 (d, *J =* 8.3 Hz, 2H), 7.43 (d, *J =* 8.1 Hz, 2H), 7.12 (d, *J =* 7.6 Hz, 2H), 6.85 (d, *J =* 5.5 Hz, 2H), 6.49 (d, *J =* 13.5 Hz, 2H), 5.80 (s, 1H), 5.18 (d, *J =* 8.2 Hz, 1H), 4.66 (s, 1H), 2.66 (t, 2H), 2.41–2.31 (m, 2H), 2.03 (s, 3H), 1.48 (s, 9H). ^13^C-NMR (CDCl_3_): δ 198.6, 170.5, 155.6, 150.4, 135.8, 131.6, 130.9, 130.4, 128.6, 122.5, 115.8, 79.4, 60.2, 51.8, 28.6, 25.1, 22.4, 9.5. ESI-MS (*m/z*): (M−H)^−^ = 586.22. Anal. Calcd. for C_29_H_33_NO_7_Se: C, 59.38; H, 5.67; N, 2.39%. Found: C, 59.32; H, 5.61; N, 2.33.

#### 3.3.2. General Procedure for the Synthesis of **10e**–**h**

To a stirred solution of **9a** or **9b** (10 mmol) in anhydrous chloroform (50 mL) was added a solution of CCM or BCM (3 mmol) in chloroform (40 mL) dropwise. After 15 min, the reaction mixture was basified with triethylamine (1 mL) and stirred for 20 min, followed by the addition of DCC (3 mmol). The reaction mixture was stirred for 8 h and monitored by TLC. Upon completion of the reaction, 1, 3-dicyclohexylurea (DCU) was removed by filtration, and the filtrate was evaporated. The resulting residue was dissolved in chloroform (50 mL), washed consecutively with 1 M HCl (50 mL), 5% NaHCO_3_ solution (50 mL), brine (50 mL) and H_2_O (50 mL), dried over anhydrous Na_2_SO_4_, filtered and concentrated *in vacuo*. Purification by column chromatography over polyamide gel (1:10, CH_3_OH/CCl_4_) gave **10e**–**h** as yellow solids.

*Curcumin-di-N-(tert-butoxycarbonyl)-methionine* (**10e**). Yield 50%. m.p. 187–188 °C. ^1^H-NMR (400 MHz, CDCl_3_): δ 7.64 (d, *J =* 15.8 Hz, 2H), 7.21–7.09 (m, 6H), 6.60 (d, *J =* 15.8 Hz, 2H), 5.89 (s, 1H), 5.25 (d, *J =* 7.7 Hz, 2H), 4.74 (d, *J =* 4.5 Hz, 2H), 3.89 (s, 6H), 2.71 (t, *J =* 7.6 Hz, 4H), 2.34 (m, 4H), 2.18 (s, 6H), 1.49 (s, 18H). ^13^C-NMR (CDCl_3_): δ 198.5, 170.3, 155.6, 151.2, 142.5, 141.3, 131.8, 130.6, 124.4, 121.9, 110.9, 79.6, 56.7, 55.9, 51.3, 30.2, 29.4, 28.2, 15.1. ESI-MS (*m/z*): (M−H)^−^ = 829.33. Anal. Calcd. for: C_41_H_54_N_2_O_12_S_2_: C, 59.26; H, 6.55; N, 3.37%. Found: C, 59.21; H, 6.51; N, 3.32%.

*Curcumin-di-N-(tert-butoxycarbonyl)-selenomethionine* (**10f**). Yield 58%. m.p. 200–201 °C. ^1^H-NMR (400 MHz, CDCl_3_): δ 7.62 (d, *J =* 15.4 Hz, 2H), 7.24–6.81 (m, 6H), 6.58 (d, *J =* 14.6 Hz, 2H), 5.87 (s, 1H), 5.19 (d, *J =* 5.7 Hz, 2H), 4.71 (s, 2H), 3.87 (s, 6H), 2.70 (t, 4H), 2.29 (m, 4H), 2.05 (s, 6H), 1.47 (s, 18H). ^13^C-NMR (CDCl_3_): δ 198.9, 170.7, 155.9, 151.8, 143.2, 141.1, 131.8, 124.4, 121.9, 110.9, 60.1, 55.8, 51.9, 28.4, 25.1, 9.4. ESI-MS (*m/z*): (M−H)^−^ = 926.08. Anal. Calcd. for: C_41_H_54_N_2_O_12_Se_2_: C, 51.58; H, 5.70; N, 3.17%. Found: C, 51.51; H, 5.73; N, 3.12%.

*Bisdesmethoxycurcumin-di-N-(tert-butoxycarbonyl)-methionine* (**10g**). Yield 65%. m.p. 207–208 °C. ^1^H-NMR (400 MHz, CDCl_3_): δ 7.64 (d, *J =* 15.8 Hz, 2H), 7.58 (d, *J =* 8.4 Hz, 4H), 7.16 (d, *J =* 8.4 Hz, 4H), 6.59 (d, *J =* 15.8 Hz, 2H), 5.84 (s, 1H), 5.20 (d, *J =* 6.2 Hz, 2H), 4.66 (s, 2H), 2.66 (t, 4H), 2.31 (m, 2H), 2.15 (s, 6H), 2.10(m, 2H), 1.47 (s, 18H). ^13^C-NMR (CDCl_3_): δ198.9, 170.2, 159.8, 150.7, 135.2, 130.6, 129.4, 121.7, 79.7, 56.9, 52.3, 31.2, 30.4, 28.6, 15.5. ESI-MS (*m/z*): (M−H)^−^ = 769.5. Anal. Calcd. for: C_39_H_50_N_2_O_10_S_2_: C, 60.76; H, 6.54; N, 3.63%. Found: C, 60.71; H, 6.50; N, 3.67%.

*Bisdesmethoxycurcumin-di-N-(tert-butoxycarbonyl)-selenomethionine* (**10h**). Yield 62%. m.p. 214–215 °C. ^1^H-NMR (400 MHz, CDCl_3_): δ 7.65 (d, *J =* 15.8 Hz, 2H), 7.58 (d, *J =* 8.6 Hz, 4H), 7.16 (d, *J =* 8.6 Hz, 4H), 6.59 (d, *J =* 15.8 Hz, 2H), 5.84 (s, 1H), 5.15 (d, *J =* 7.0 Hz, 2H), 4.65 (s, 2H), 2.67 (t, *J =* 7.4 Hz, 4H), 2.44–2.29 (m, 4H), 2.34 (d, *J =* 18.5 Hz, 2H), 2.27–2.08 (m, 4H), 2.04 (s, 6H), 1.47 (s, 18H). ^13^C-NMR (CDCl_3_): δ 198.8, 170.2, 156.2, 150.7, 135.2, 130.5, 129.7, 122.2, 79.5, 60.3, 52.4, 28.7, 25.2, 22.3, 9.3. ESI-MS (*m/z*): (M−H)^−^ = 863.3. Anal. Calcd. for C_39_H_50_N_2_O_10_Se_2_: C, 54.17; H, 5.83; N, 3.24%. Found: C, 54.11; H, 5.89; N, 3.20%.

#### 3.3.3. General Procedure for the Synthesis of Compounds **11a**–**h**

A solution of HCl/EA (4 mL, 4 M) in a 25-mL round-bottom flask equipped with a magnetic stir-bar was cooled by an ice-water bath under nitrogen. Compound (**10a**–**h**, 1 mmol) was added in one portion with stirring. The ice-bath was removed, and the mixture was stirred at 25 °C. After 30 min, TLC indicated that the reaction was completed. The product separated as a precipitate, as it was insoluble in EA.

*Curcumin-mono-methionine* (**11a**). Yield 60%. m.p. 60–61 °C. ^1^H-NMR (400 MHz, DMSO): δ 7.63 (d, *J =* 7.6 Hz, 1H), 7.59 (d, *J =* 7.9 Hz, 1H), 7.40–7.24 (m, 4H), 7.18 (d, *J =* 8.2 Hz, 1H), 7.00 (d, *J =* 16.0 Hz, 1H), 6.85 (d, *J =* 8.2 Hz, 1H), 6.81 (d, *J =* 15.9 Hz, 1H), 6.15 (s, 1H), 3.87 (s, 3H), 3.84 (s, 3H), 2.78–2.71 (m, 2H), 2.28–2.23 (m, 2H), 2.12 (s, 3H). ^13^C-NMR (CDCl_3_): δ 198.7, 168.2, 151.7, 149.2, 147.5, 142.9, 141.4, 131.9, 130.5, 124.7, 122.9, 121.8, 117.2, 56.4, 52.3, 33.9, 29.7, 15.5. ESI-MS (*m/z*): (M−H)^−^ = 498.17. Anal. Calcd. for C_26_H_29_NO_7_S: C, 62.51; H, 5.85; N, 2.80%. Found: C, 62.58; H, 5.80; N, 2.83%.

*Curcumin-mono-selenomethionine* (**11b**). Yield 65%. m.p. 69–70 °C. ^1^H-NMR (400 MHz, DMSO): δ 7.63 (d, *J =* 7.7 Hz, 1H), 7.59 (d, *J =* 7.6 Hz, 1H), 7.41–7.23 (m, 4H), 7.18 (d, *J =* 8.3 Hz, 1H), 7.01 (d, *J =* 16.0 Hz, 1H), 6.85 (d, *J =* 8.2 Hz, 1H), 6.81 (d, *J =* 15.8 Hz, 1H), 6.15 (s, 1H), 3.87 (s, 3H), 3.84 (s, 3H), 2.78–2.69 (m, 2H), 2.33 (m, 2H), 2.02 (s, 3H). ^13^C-NMR (CDCl_3_): δ198.5, 167.8, 151.4, 149.2, 147.5, 142.9, 141.2, 131.7, 130.2, 123.1, 122.2, 120.8, 116.2, 55.8, 51.8, 32.8, 25.7, 9.4. ESI-MS (*m/z*): (M−H)^−^ = 546.1. ESI-MS (m/z): (M−H)^−^ = 546.1. Anal. Calcd. for C_26_H_29_NO_7_Se: C, 62.51; H, 5.85; N, 2.80%. Found: C, 62.58; H, 5.80; N, 2.83%.

*Bisdesmethoxycurcumin-mono-methionine* (**11c**). Yield 62%. m.p. 73–74 °C. ^1^H-NMR (400 MHz, DMSO): δ 7.84 (d, *J =* 8.7 Hz, 2H), 7.59 (d, *J =* 8.5 Hz, 2H), 7.34 (d, *J =* 8.7 Hz, 2H), 6.94 (d, *J =* 16.0 Hz, 1H), 6.85 (d, *J =* 8.6 Hz, 2H), 6.74 (d, *J =* 15.9 Hz, 1H), 6.14 (s, 1H), 4.40 (s, 2H), 2.69 (m, 2H), 2.37–2.21 (m, 2H), 2.11 (s, 3H). ^13^C-NMR (CDCl_3_): δ 198.9, 168.0, 157.4, 150.6, 135.1, 130.7, 130.5, 121.1, 115.2, 52.2, 34.1, 30.7, 15.4. ESI-MS (*m/z*): (M−H)^−^ = 438.17. Anal. Calcd. for C_24_H_25_NO_5_S: C, 65.58; H, 5.73; N, 3.19%. Found: C, 65.53; H, 5.76; N, 3.21%.

*Bisdesmethoxycurcumin-mono-selenomethionine* (**11d**). Yield 65%. m.p. 77–78 °C. ^1^H-NMR (400 MHz, DMSO): δ 7.84 (d, *J =* 8.6 Hz, 2H), 7.59 (d, *J =* 8.3 Hz, 2H), 7.33 (d, *J =* 8.6 Hz, 2H), 6.95 (d, *J =* 15.9 Hz, 1H), 6.85 (d, *J =* 8.5 Hz, 2H), 6.74 (d, *J =* 15.9 Hz, 1H), 6.14 (s, 1H), 4.40 (s, 1H), 2.75 (t, *J =* 8.8 Hz, 2H), 2.38–2.28 (m, 2H), 2.01 (s, 3H). ^13^C-NMR (CDCl_3_): δ198.7, 168.5, 157.6, 150.8, 136.3, 130.8, 130.4, 121.3, 115.2, 55.5, 51.9, 34.1, 25.7, 9.4. ESI-MS (*m/z*): (M−H)^−^ = 486.08. Anal. Calcd. for C_24_H_25_NO_5_Se: C, 59.26; H, 5.18; N, 2.88%. Found: C, 59.23; H, 5.16; N, 2.92%.

*Curcumin-di-methionine* (**11e**). Yield 70%. m.p. 82–83 °C. ^1^H-NMR (400 MHz, DMSO): δ 7.68 (d, *J =* 16.0 Hz, 2H), 7.60 (s, 2H), 7.41 (d, *J =* 7.9 Hz, 2H), 7.06 (d, *J =* 15.9 Hz, 2H), 6.24 (s, 1H), 4.44 (s, 2H), 3.87 (s, 6H), 2.82–2.69 (m, 4H), 2.32–2.24 (m, 4H), 2.12 (s, 6H). ^13^C-NMR (CDCl_3_): δ 197.7, 168.2, 151.6, 142.8, 141.3, 132.3, 130.6, 122.4, 120.3, 111.2, 56.5, 52.3, 33.9, 30.3, 15.7. ESI-MS (*m/z*): (M−H)^−^ = 629.25. Anal. Calcd. for C_31_H_38_N_2_O_8_S_2_: C, 59.03; H, 6.07; N, 4.44%. Found: C, 59.07; H, 6.11; N, 4.45%.

*Curcumin-di-selenomethionine* (**11f**). Yield: 75%. m.p. 88–89 °C. ^1^H-NMR (400 MHz, DMSO): δ 7.68 (d, *J =* 15.9 Hz, 2H), 7.60 (s, 2H), 7.40 (d, *J =* 7.8 Hz, 2H), 7.28 (d, *J =* 8.2 Hz, 2H), 7.06 (d, *J =* 16.0 Hz, 2H), 6.23 (s, 1H), 4.44 (s, 2H), 3.87 (s, 6H), 2.80–2.69 (m, 4H), 2.35–2.26 (m, 4H), 2.02 (s, 6H). ^13^C-NMR (CDCl_3_): δ 198.2, 168.1, 151.1, 143.1, 141.2, 131.8, 130.5, 124.4, 121.3, 110.2, 56.5, 52.3, 25.3, 9.2. ESI-MS (*m/z*): (M–H)^−^ = 725.17. Anal. Calcd. for C_31_H_38_N_2_O_8_Se: C, 51.39; H, 5.29; N, 3.87%. Found: C, 51.37; H, 5.25; N, 3.84%.

*Bisdesmethoxycurcumin-di-methionine* (**11g**). Yield 77%. M.p. 92–93 °C. ^1^H-NMR (400 MHz, DMSO): δ 7.86 (d, *J =* 8.5 Hz, 4H), 7.67 (d, *J =* 16.0 Hz, 2H), 7.35 (d, *J =* 8.5 Hz, 4H), 6.97 (d, *J =* 15.7 Hz, 2H), 6.18 (s, 1H), 4.40 (s, 2H), 2.78–2.69 (m, 4H), 2.27 (d, 4H), 2.11 (s, 6H). ^13^C-NMR (CDCl_3_): δ 198.8, 168.3, 150.5, 134.8, 130.8, 121.7, 121.8, 52.5, 33.8, 29.3, 15.4. ESI-MS (*m/z*): (M−H)^−^ = 570.25. Anal. Calcd. for C_29_H_34_N_2_O_6_S_2_: C, 61.03; H, 6.00; N, 4.91%. Found: C, 61.11; H, 6.07; N, 4.92%.

*Bisdesmethoxycurcumin-di-selenomethionine* (**11h**). Yield 70%. m.p. 97–98 °C. ^1^H-NMR (400 MHz, DMSO): δ 7.86 (d, *J =* 8.6 Hz, 4H), 7.68 (d, *J =* 11.5 Hz, 2H), 7.35 (d, *J =* 8.6 Hz, 4H), 6.99 (d, *J =* 16.0 Hz, 2H), 6.23 (s, 1H), 4.40 (t, *J =* 6.2 Hz, 2H), 2.81–2.68 (m, 4H), 2.41–2.24 (m, 4H), 2.01 (s, 6H). ^13^C-NMR (CDCl_3_): δ 198.8, 168.2, 150.7, 135.0, 130.9, 129.8, 121.7, 55.8, 52.2, 25.3, 9.4. ESI-MS (*m/z*): (M−H)^−^ = 665.00. Anal. Calcd. for C_29_H_34_N_2_O_6_Se_2_: C, 52.42; H, 5.16; N, 4.20%. Found: C, 52.49; H, 5.14; N, 4.23%.

### 3.4. General Method for the Synthesis of Curcuminoid-Caffeic Acid and Curcuminoid-Ferulic Acid Conjugates

#### 3.4.1. General Procedure for the Synthesis of **13a**–**b**

**12a** or **12b** (1 mmol) was heated under reflux with Ac_2_O (20 mmol) for 5 h. Then, acetic acid solution was added to the still hot solution. The product, crystallized upon cooling, was filtered off and washed with cold H_2_O and Et_2_O and dried *in vacuo* to give **13a** or **13b** as a white solid.

*Ferulic acid acetate* (**13a**). Yield 80%. ^1^H-NMR (400 MHz, CDCl_3_): δ 7.60 (d, *J* = 24 Hz, 1H), 7.01 (m, 3H). 6.30 (d, *J* = 24 Hz, 1H), 3.90 (s, 3H), 2.33(s, 3H). ESI-MS (*m/z*): (M−H)^−^ = 236.08. Anal. Calcd. for C_12_H_12_O_5_: C, 61.01; H, 5.12; O, 33.87%. Found: C, 61.06; H, 5.16; O, 33.82%.

*Caffeic acid acetate* (**13b**). Yield 85%. ^1^H-NMR (400MHz, CDCl_3_): δ 7.47 (d, *J* = 15.8 Hz, 1H), 7.33 (m, 3H), 6.33(d, *J* = 16.0 Hz, 1H), 2.30(s, 3H), 2.28(s, 3H). ESI-MS (*m/z*): (M−H)^−^ = 263.52. Anal. Calcd. for C_13_H_12_O_6_: C, 59.09; H, 4.58; O, 36.33%. Found: C, 59.12; H, 4.52; O, 36.30%.

#### 3.4.2. General Procedure for the Synthesis of **9a**–**d**

To a solution of **13a** or **13b** (1 mmol) in chloroform (50 mL), EDCI (1.2 mmol), HOBT (1.2 mmol) and DIEA (1 mL) were added. After stirring at 0 °C for 1 h, a chloroform solution (40 mL) of CCM or BCM (1 mmol) was added dropwise to the mixtures, which was allowed to be stirred overnight at room temperature. After completion of the reaction, as indicated by TLC, the mixture was washed with hydrochloric acid (1 M) and then with brine. The organic layer was dried with anhydrous Na_2_SO_4_ and evaporated to dryness under vacuum. Purification by dry column flash chromatography on silica gel-G (CHCl_2_:MeOH = 1:10) gave **14a**–**d** as a yellow solid.

*Curcumin-mono-ferulic acid acetate* (**14a**). Yield 57%. 183–184 °C. ^1^HNMR (400 MHz, CDCl_3_): δ 7.60 (d, *J =* 20.2 Hz, 1H),7.45 (d, *J =* 15.8 Hz, 1H), 7.10 (d, *J =* 2.5Hz, 1H), 7.05-6.92 (m, 6H), 7.01 (m, 3H), 6.92–6.72 (m, 2H), 6.30 (d, *J =* 20.0 Hz,1H), 6.20(d, *J =* 16.0 Hz, 1H), 5.50 (s, 1H), 3.90 (s, 3H), 3.91 ( s, 3H), 3.89 (s, 3H,), 3.87 (s, 3H), 2.32(s, 3H). ^13^C-NMR (CDCl_3_): δ 198.6, 169.0, 164.3, 151.2, 149.5, 147.6, 142.7, 137.5, 130.6, 128.3, 123.2, 122.5, 121.2, 116.8, 115.0, 111.2, 110.9, 55.9, 52.1, 20.1. ESI-MS (*m/z*): (M−H)^−^ = 553.5. Anal. Calcd. for C_33_H_30_O_10_: C, 67.57; H, 5.15; O, 27.28%. Found: C, 67.50; H, 5.18; O, 27.30%.

*Curcumin-mono-caffeic acid acetate* (**14b**). Yield 60%. m.p. 179–180 °C. ^1^H-NMR (400 MHz, CDCl_3_): δ 7.65 (d, *J =* 6.0 Hz, 1H), 7.55 (d, *J =* 6.0 Hz, 1H), δ7.45 (d, *J =* 15.8 Hz, 1H), 7.33 (m, 3H), 7.21–7.09 (m, 4H), 7.06 (d, *J =* 1.8 Hz, 1H), 6.94 (d, *J =* 8.2 Hz, 1H), 6.58 (d, *J =* 15.8 Hz, 1H), 6.50 (d, *J =* 15.8 Hz, 1H), 6.33 (d, *J =* 16.0 Hz, 1H), 5.83 (s, 1H), 3.90 (s, 3H), 3.88 (s, 3H), 2.30 (s, 3H), 2.28 (s, 3H). ^13^C-NMR (CDCl_3_): δ 198.6, 169.2, 164.8, 151.7, 150.0, 148.1, 142.9, 137.8, 130.6, 128.2, 123.9, 122.8, 121.6, 117.2, 115.6, 111.8, 110.6, 55.9, 52.1, 20.5. ESI-MS (*m/z*): (M−H)^−^ = 613.18. Anal. Calcd. for C_34_H_30_O_11_: C, 66.44; H, 4.92; O, 28.64%. Found: C, 66.40; H, 4.88; O, 28.60%.

*Bisdesmethoxycurcumin-mono-ferulic acid acetate* (**14c**). Yield 55%. m.p. 197–198 °C. ^1^H-NMR (400 MHz, CDCl_3_): δ 7.67 (d, *J =* 15.5 Hz, 2H), 7.62 (d, *J =* 20.4 Hz, 1H), 7.57 (d, *J =* 8.6 Hz, 2H), 7.46 (d, *J =* 8.6 Hz, 2H), 7.12 (d, *J =* 7.6 Hz, 2H), 7.01 (m, 3H), 6.85 (d, *J =* 7.5 Hz, 2H), 6.49 (d, *J =* 13.5 Hz, 2H), 6.33 (d, *J =* 20.6 Hz, 1H), 5.80 (s, 1H), 3.90 (s, 3H), 2.31(s, 3H). ^13^C-NMR (CDCl_3_): δ 198.7, 169.0, 164.2, 157.6, 151.8, 150.6, 148.1, 141.3, 135.3, 132.0, 130.6, 124.8, 121.7, 115.8, 110.5, 110.5, 55.9, 52.2, 20.4. ESI-MS (*m/z*): (M−H)^−^ = 525.56. Anal. Calcd. for C_31_H_26_O_8_: C, 70.71; H, 4.98; O, 24.31%. Found: C, 70.65; H, 5.02; O, 24.36%.

*Bisdesmethoxycurcumin-mono-caffeic acid acetate* (**14d**). Yield 55%. m.p. 193–194 °C. ^1^H-NMR (400 MHz, CDCl_3_): δ 7.61 (d, *J =* 12.9 Hz, 2H), 7.54 (d, *J =* 8.3 Hz, 2H), δ7.49 (d, *J =* 15.8 Hz, 1H), 7.43 (d, *J =* 8.1 Hz, 2H), 7.12 (d, *J =* 8.5 Hz, 2H), 7.36 (m, 3H), 6.85 (d, *J =* 7.8 Hz, 2H), 6.56 (d, *J =* 15.8 Hz, 1H), 6.48 (d, *J =* 15.8 Hz, 1H), 6.38(d, *J =* 16.0 Hz, 1H), 5.80 (s, 1H), 2.32(s, 3H), 2.30(s, 3H). ^13^C-NMR (CDCl_3_): δ 198.7, 168.8, 164.2, 157.5, 150.7, 147.8, 142.6, 135.1, 130.6, 130.2, 126.3, 122.9, 121.5, 115.8, 115.2, 53.3, 20.7. ESI-MS (*m/z*): (M−H)^−^ = 553.10. Anal. Calcd. for C_3__2_H_26_O_9_: C, 69.31; H, 4.73; O, 25.97%. Found: C, 69.35; H, 4.76; O, 25.94%.

#### 3.4.3. General Procedure for the Synthesis of **14e**–**h**

To a stirred, ice-cooled, solution of **13a** or **13b** (20 mL, 10 mmol), a solution of CCM or BCM (20 mmol) in dry CHCl_3_ (30 mL) was added dropwise, and the resulting solution was stirred for 10 min at 0 °C. Then, TEA (10 mmol) was added to the solution and stirred at room temperature for 10 h. After completion of the reaction, as indicated by TLC, the mixture was washed with sodium bicarbonate (1 M) and then with brine. The organic layer was dried with anhydrous Na_2_SO_4_ and evaporated to dryness under vacuum. Purification by chromatography on silica (CHCl_3_:MeOH = 20:1) gave **14e**–**h** as a yellow solid.

*Curcumin-di-ferulic acid acetate* (**14e**). Yield 60%. m.p. 206–207 °C. ^1^HNMR (400 MHz, CDCl_3_): δ 7.69 (d, *J =* 15.8 Hz, 2H), 7.64 (d, *J =* 20.8 Hz, 1H), 7.31–7.13 (m, 6H), 7.09 (m, 6H), 6.65 (d, *J =* 15.8 Hz, 2H), 5.89 (s, 1H), 6.30 (d, *J =* 20.0 Hz, 2H), 3.90 (s, 6H), 2.33(s, 6H), 2.31(s, 6H). ^13^C-NMR (CDCl_3_): δ198.9, 169.0, 164.3, 151.5, 147.8, 142.5, 141.2, 137.4, 131.6, 130.4, 124.5, 121.9, 115.5, 110.8, 55.9, 51.9, 20.2. ESI-MS (*m/z*): (M−H)^−^ = 803.45. Anal. Calcd. for C_45_H_40_O_14_: C, 67.16; H, 5.01; O, 27.83%. Found: C, 67.20; H, 5.07; O, 27.80%.

*Curcumin-di-caffeic acid acetate* (**14f**). Yield 57%. m.p. 201–202 °C. ^1^HNMR (400 MHz, CDCl_3_): ^1^H-NMR (400 MHz, CDCl_3_) δ 7.66 (d, *J =* 16.2 Hz, 2H), δ7.47 (d, *J =* 15.8 Hz, 2H), 7.36–7.25 (m, 6H), 7.21–7.10 (m, 6H), 6.60 (d, *J =* 16.0 Hz, 2H), 6.33(d, *J =* 16.0 Hz, 2H), 3.89 (s, 6H), 2.30 (s, 6H), 2.28 (s, 6H). ^13^C-NMR (CDCl_3_): δ 198.9, 169, 164.3, 151.5, 147.9, 143.2, 142.6, 142.2, 137.4, 131.5, 131.0, 130.6, 126.8, 124.6, 123.8, 122.8, 121.2, 115.1, 110.7, 55.8, 51.8, 20.3. ESI-MS (*m/z*): (M−H)^−^ = 859.25. Anal. Calcd. for C_47_H_40_O_16_: C, 65.58; H, 4.68; O, 29.74%. Found: C, 65.50; H, 4.62; O, 29.70%.

*Bisdesmethoxycurcumin-di-ferulic acid acetate* (**14g**). Yield 55%. m.p. 217–218 °C. ^1^H-NMR (400 MHz, CDCl_3_): δ 7.68 (d, *J =* 15.8 Hz, 2H), 7.63 (d, *J =* 21.0 Hz, 2H), 7.60 (d, *J =* 8.5 Hz, 4H), 7.21 (d, *J =* 8.6 Hz, 4H), 7.05 (m, 6H), 6.62 (d, *J =* 15.8 Hz, 2H), 6.30 (d, *J =* 20.8 Hz, 1H), 5.86 (s, 1H), 3.91 (s, 6H), 2.33(s, 6H). ^13^C-NMR (CDCl_3_): δ 198.9, 169.3, 164.3, 151.5, 150.5, 147.9, 141.2, 134.8, 131.6, 130.7, 130.4, 129.6, 129.6, 124.4, 121.9, 121.5, 121.5, 115.5, 110.9, 55.8, 51.9, 20.4. ESI-MS (*m/z*): (M−H)^−^ = 743.21. Anal. Calcd. for C_43_H_36_O_12_: C, 69.35; H, 4.87; O, 25.78%. Found: C, 69.40; H, 4.80; O, 25.82%.

*Bisdesmethoxycurcumin-di-caffeic acid acetate* (**14h**). Yield 65%. m.p. 213–214 °C. ^1^HNMR (400 MHz, CDCl_3_): δ 7.68 (d, *J =* 15.8 Hz, 2H), 7.59 (d, *J =* 8.5 Hz, 4H), 7.50(d, *J =* 16.0 Hz, 2H), 7.35–7.25 (m, 6H), 7.20 (d, *J =* 8.5 Hz, 4H), 6.61 (d, *J =* 16.2 Hz, 2H), 6.30 (d, *J =* 16.2 Hz, 2H), 2.34 (s, 6H), 2.32 (s, 6H). ^13^C-NMR (CDCl_3_): δ 198.9, 169.3, 164.3, 150.5, 147.9, 143.2, 142.5, 134.9, 131.2, 130.7, 130.4, 129.6, 126.4, 123.8, 122.9, 121.5, 121.5, 115.5, 51.9, 20.3. ESI-MS (*m/z*): (M−H)^−^ = 799.20. Anal. Calcd. for C_45_H_36_O_14_: C, 67.50; H, 4.53; O, 27.97%. Found: C, 67.55; H, 4.50; O, 27.92%.

#### 3.4.4. General Procedure for the Synthesis of **15a**–**h**

A solution of **14a**–**h** (1 mmol) and CH_3_ONa (10 mmol) in MeOH (10 mL) was stirred at room temperature overnight. The solvent was evaporated and the residue diluted with water and HCl (1 M) and extracted with CHCl_3_. The organic layers were dried, and the solvent was evaporated *in vacuo*. The residue was purified by flash chromatography on silica (CHCl_3_:MeOH = 10:1) to give **15a**–**h** as a yellow powder.

*Curcumin-mono-ferulic acid* (**15a**): Yield 65%. m.p 143–144 °C. ^1^H-NMR (400 MHz, CDCl_3_): δ 7.66 (d, *J =* 8.0 Hz, 1H), 7.60 (d, *J =* 8.2 Hz, 1H), 7.57 (d, *J =* 19.5 Hz, 1H), 7.38–7.26 (m, 4H), 7.19 (d, *J =* 8.5 Hz, 1H), 7.03 (m, 3H), 7.07 (d, *J =* 16.0 Hz, 1H), 6.85 (d, *J =* 8.2 Hz, 1H), 6.81 (d, *J =* 15.8 Hz, 1H), 6.36 (d, *J =* 20.0 Hz, 1H), 3.92 (s, 3H), 3.87 (s, 3H). ^13^C-NMR (CDCl_3_): δ 198.8, 164.5, 151.2, 149.1, 147.5, 138.2, 132.2, 132.2, 130.4, 126.8, 123.8, 121.6, 116.8, 115.8, 112.2, 56.4, 52.6. ESI-MS (*m/z*): (M−H)^−^ = 543.10. Anal. Calcd. for C_31_H_28_O_9_: C, 68.37; H, 5.18; O, 26.44%. Found: C, 68.40; H, 5.20; O, 26.50%.

*Curcumin-mono-caffeic acid* (**15b**): Yield 60%. m.p. 135–136 °C. ^1^H-NMR (400MHz, CDCl_3_): δ 7.66 (d, *J =* 7.5 Hz, 1H), 7.58 (d, *J =* 8.0 Hz, 1H), 7.49 (d, *J =* 15.8 Hz, 1H), 7.38–7.26 (m, 4H), 7.35 (m, 3H), 7.20(d, *J =* 8.0 Hz, 1H), 7.04 (d, *J =* 16.0 Hz, 1H), 6.88 (d, *J =* 8.2 Hz, 1H), 6.85 (d, *J =* 15.9 Hz, 1H), 6.38 (d, *J =* 16.0 Hz, 1H), 3.90 (s, 3H). ^13^C-NMR (CDCl_3_): δ 198.9, 164.3, 150.8, 147.9, 146.5, 145.9, 142.8, 139.2, 132.2, 131.2, 128.4, 125.8, 123.5, 122.6, 116.8, 115.5, 115.0, 114.2, 51.6. ESI-MS (*m/z*): (M−H)^−^ = 529.12. Anal. Calcd. for C_30_H_26_O_9_: C, 67.92; H, 4.94; O, 27.14%. Found: C, 67.90; H, 4.90; O, 27.10%.

*Bisdesmethoxycurcumin-mono-ferulic acid* (**15c**). Yield 65%. m.p. 155–156 °C. ^1^H-NMR (400 MHz, CDCl_3_): δ 7.88 (d, *J =* 8.5 Hz, 2H), 7.63(d, *J =* 8.6Hz, 2H), 7.60 (d, *J =* 20.0 Hz, 1H), 7.38 (d, *J =* 8.6 Hz, 2H), 7.01 (m, 3H), 6.98 (d, *J =* 15.9 Hz, 1H), 6.89 (d, *J =* 8.6 Hz, 2H), 6.78 (d, *J =* 16.0 Hz, 1H), 6.30 (d, *J =* 20.6 Hz, 1H). ^13^C-NMR (CDCl_3_): δ198.5, 164.3, 157.3, 150.5, 149.4, 147.3, 130.8, 130.5, 121.5, 115.9, 111.6, 56.3, 52.5. ESI-MS (*m/z*): (M−H)^−^ = 483.19. Anal. Calcd. for C_29_H_24_O_7_: C, 71.89; H, 4.99; O, 23.12%. Found: C, 71.80; H, 4.90; O, 23.14%.

*Bisdesmethoxycurcumin-mono-caffeic acid* (**15d**). Yield 65%. m.p 148–149 °C. ^1^H-NMR (400 MHz, CDCl_3_): δ 7.86 (d, *J =* 8.5 Hz, 2H), 7.62 (d, *J =* 8.5 Hz, 2H), 7.48 (d, *J =* 15.8 Hz, 1H), 7.38 (d, *J =* 8.5 Hz, 2H), 7.35-7.28 (m, 3H), 6.94 (d, *J =* 16.0 Hz, 1H), 6.85 (d, *J =* 8.6 Hz, 2H), 6.74 (d, *J =* 15.9 Hz, 1H), 6.36 (d, *J =* 16.0 Hz, 1H). ^13^C-NMR (CDCl_3_): δ 198.9, 164.3, 157.9, 150.4, 147.98, 146.3, 145.8, 134.6, 130.4, 123.5, 121.3, 117.5, 115.9, 51.6. ESI-MS (*m/z*): (M−H)^−^ = 469.39. Anal. Calcd. for C_28_H_22_O_7_: C, 71.48; H, 4.71; O, 23.81%. Found: C, 71.40; H, 4.80; O, 23.94%.

*Curcumin-di-ferulic acid* (**15e**). Yield 60%. m.p. 166–167 °C. ^1^H-NMR (400 MHz, CDCl_3_): δ 7.66 (d, *J =* 15.9 Hz, 2H), 7.60 (s, 2H), 7.56 (d, *J =* 23.8 Hz, 2H), 7.42 (d, *J =* 8.0 Hz, 2H), 7.32 (d, *J =* 8.2 Hz, 2H), 7.05 (m, 6H), 7.01 (d, *J =* 16.0 Hz, 2H), 6.30 (d, *J =* 23.8 Hz, 2H), 3.90 (s, 6H), 3.88 (s, 3H), 3.87 (s, 3H). ^13^C-NMR (CDCl_3_): δ 198.9, 164.5, 151.5, 149.2, 147.8, 142.5, 137.5, 131.6, 127.6, 124.4, 122.5, 121.4, 116.9, 109.6, 55.6, 52.1. ESI-MS (*m/z*): (M−H)^−^ = 719.20. Anal. Calcd. for C_39_H_32_O_12_: C, 68.33; H, 5.03; O, 26.64%. Found: C, 68.40; H, 5.00; O, 26.70%.

*Curcumin-di-caffeic acid* (**15f**). Yield 62%. m.p. 162–163 °C. ^1^H-NMR (400 MHz, CDCl_3_): δ 7.69 (d, *J =* 16.0 Hz, 2H), 7.49 (d, *J =* 15.8 Hz, 2H), 7.60 (d, *J =* 8.5 Hz, 2H), 7.46 (d, *J =* 7.9 Hz, 2H), 7.38 (m, 6H), 6.38 (d, *J =* 16.0 Hz, 2H), 7.09 (d, *J =* 15.9 Hz, 2H), 3.90 (s, 6H). ^13^C-NMR (CDCl_3_): δ 198.9, 164.2, 151.7, 148.4, 146.4, 143.3, 137.9, 132.4, 130.6, 128.5, 124.6, 123.2, 121.9, 115.3, 110.5, 55.5, 51.5. ESI-MS (*m/z*): (M−H)^−^ = 743.25. Anal. Calcd. for C_39_H_32_O_12_: C, 67.63; H, 4.66; O, 27.72%. Found: C, 67.70; H, 4.70; O, 28.10%.

*Bisdesmethoxycurcumin-di-ferulic acid* (**15g**). Yield 57%. m.p. 191-192 °C. ^1^H-NMR (400 MHz, CDCl_3_): δ 7.88 (d, *J =* 8.6 Hz, 4H), 7.70 (d, *J =* 11.5 Hz, 2H), 7.60 (d, *J =* 20.0 Hz, 2H), 7.38 (d, *J =* 8.5 Hz, 4H), 7.25–7.13 (m, 6H), 6.96 (d, *J =* 16.0 Hz, 2H), 6.36 (d, *J =* 20.2 Hz, 1H), 3.90 (s, 6H). ^13^C-NMR (CDCl_3_): 198.9, 168.5, 148.7, 147.5, 134.6, 130.2, 129.5, 127.6, 123.3, 121.5, 116.8, 115.9, 149.2, 56.6, 52.2. ESI-MS (*m/z*): (M−H)^−^ = 659.10. Anal. Calcd. for C_39_H_32_O_10_: C, 70.90; H, 4.88; O, 24.22%. Found: C, 70.85; H, 4.90; O, 24.30%.

*Bisdesmethoxycurcumin-di-caffeic acid* (**15h**). Yield 55%. m.p. 181–182 °C. ^1^H-NMR (400 MHz, CDCl_3_): δ 7.85 (d, *J =* 8.6 Hz, 4H), 7.69 (d, *J =* 16.0 Hz, 2H), 7.49 (d, *J =* 16.2 Hz, 2H), 7.35 (d, *J =* 8.6 Hz, 4H), 7.30-7.18 (m, 6H), 6.97 (d, *J =* 15.7 Hz, 2H), 6.37 (d, *J =* 16.4 Hz, 2H). ^13^C-NMR (CDCl_3_): 198.9, 150.4, 146.5, 145.5, 139.5, 130.3, 129.8, 128.4, 123.5, 121.5, 117.5, 115.3, 51.5. ESI-MS (*m/z*): (M−H)^−^ = 631.10. Anal. Calcd. for C_37_H_28_O_10_: C, 70.25; H, 4.46; O, 25.29%. Found: C, 70.30; H, 4.50; O, 25.30%.

### 3.5. General Procedure for the Synthesis of Curcuminoid-Glycyrrhetinic Acid Conjugates

Glycyrrhetinic acid (**16**, 1 mmol) was heated under reflux with Ac_2_O (50 mmol) for 2 h. Then, acetic acid solution was added to the still hot solution. The product, crystallized upon cooling, was filtered off and washed with cold H_2_O and Et_2_O and dried *in vacuo* to give **17** as a white solid. 

*Glycyrrhetinic acid acetate* (**17**). Yield 79%. m.p. 317–318 °C. ^1^H-NMR (400 MHz, CDCl_3_): δ 5.76 (s, 1H), 4.55 (dd, *J =* 11.8, 5.4 Hz, 1H), 2.85 (d, *J =* 13.9 Hz, 1H), 2.35 (s, 1H), 2.17 (d, *J =* 13.1 Hz, 1H), 2.07(s, 3H), 2.09–1.57 (m, 12H), 1.45–1.01 (m, 7H), 1.35 (s, 3H), 1.25(s, 3H), 1.22(s, 3H), 1.15 (s, 3H), 0.88 (s, 6H), 0.84 (s, 3H). ESI-MS (*m/z*): (M−H)^−^ = 511.65. Anal. Calcd. for C_32_H_48_O_5_: C, 74.96; H, 9.44; O, 15.60%. Found: C, 74.95; H, 9.50; O, 15.55%.

#### 3.5.1. General Procedure for the Synthesis of Compounds **18a**–**b**

To the solution of **17** (1 mmol) in chloroform (50 mL), EDCI (1.2 mmol), HOBT (1.2 mmol) and DIEA (1 mL) were added. After stirring at 0 °C for 1 h, a chloroform solution (40 mL) of CCM (1 mmol) or BCM (1 mmol) was added dropwise to the mixtures, which was allowed to be stirred overnight at room temperature. After completion of the reaction, as indicated by TLC, the mixture was washed with hydrochloric acid (1 M) and then with brine. The organic layer was dried with anhydrous Na_2_SO_4_ and evaporated to dryness under vacuum. Purification by dry column flash chromatography on silica gel-G (EA:PE = 1:5) gave **18a**–**b** as a yellow solid.

*Curcumin-mono-glycyrrhetinic acid acetate* (**18a**). Yield 55%. m.p. 225–226 °C. ^1^H-NMR (400 MHz, CDCl_3_): δ **7**.63 (d, *J =* 5.6 Hz, 1H), 7.59 (d, *J =* 5.6 Hz, 1H), 7.25–7.07 (m, 4H), 7.09 (d, *J =* 2.0 Hz, 1H), 6.96 (d, *J =* 8.4 Hz, 1H), 6.59 (d, *J =* 16.2 Hz, 1H), 6.49 (d, *J =* 15.8 Hz, 1H), 5.79 (s, 1H), 4.55 (dd, *J =* 12.0, 5.8 Hz, 1H), 3.90(s, 3H), 3.88(s, 3H), 2.85 (d, *J =* 14.4 Hz, 1H), 2.38(s, 1H), 2.23(s, 3H), 2.18 (d, *J =* 13.1 Hz, 1H), 2.09–1.57 (m, 12H), 1.46–1.03 (m,7H), 1.35 (s, 3H), 1.27(s, 3H), 1.24(s, 3H), 1.17 (s, 3H), 0.89 (s, 6H), 0.85 (s, 3H). ^13^C-NMR (CDCl_3_): δ 200.9, 176.3, 170.6, 170.2, 151.5, 149.1, 147.9, 142.8, 141.5, 131.8, 130.6, 128.3, 127.8, 124.4, 122.5, 121.6, 116.8, 111.9, 110.9, 80.6, 61.3, 56.1, 55.9, 51.8, 48.2, 45.6, 43.6, 41.9, 41.1, 37.6, 37.1, 36.6, 35.7, 32.5, 32.1, 30.5, 28.3, 26.9, 26.7, 26.2, 25.7, 23.8, 23.6, 21.5, 18.4, 17.2, 16.8. ESI-MS (*m/z*): (M−H)^−^ = 861.40. Anal. Calcd. for C_53_H_66_O_10_: C, 73.75; H, 7.71; O, 18.54%. Found: C, 73.78; H, 7.80; O, 18.59%.

*Bisdesmethoxycurcumin-mono-glycyrrhetinic acid acetate* (**18b**). Yield 55%. m.p. 232–233 °C. ^1^H-NMR (400 MHz, CDCl_3_): δ 7.69 (d, *J =* 15.9 Hz, 2H), 7.58 (d, *J =* 8.6 Hz, 2H), 7.48 (d, *J =* 8.6 Hz, 2H), 7.17 (d, *J =* 8.6 Hz, 2H), 6.89 (d, *J =* 7.8 Hz, 2H), 6.57 (d, *J =* 15.8 Hz, 1H), 6.45(d, *J =* 15.8 Hz, 1H), δ 5.79 (s, 1H), 4.58 (dd, *J =* 12.2, 6.0 Hz, 1H), 2.87 (d, *J =* 14.2 Hz, 1H), 2.36 (s, 1H), 2.25(s, 3H), 2.19 (d, *J =* 13.8 Hz, 1H), 2.09–1.59 (m, 12H), 1.48–1.04 (m, 7H), 1.37 (s, 3H), 1.26 (s, 3H), 1.23(s, 3H), 1.17 (s, 3H), 0.88 (s, 6H), 0.86 (s, 3H). ^13^C-NMR (CDCl_3_): δ 200.8, 176.3, 170.6, 170.2, 151.5, 149.1, 147.9, 142.8, 141.5, 131.8, 130.6, 128.3, 127.5, 124.3, 122.2, 121.4, 116.7, 111.6, 110.5, 80.3, 61.2, 55.5, 51.5, 48.1, 45.3, 43.2, 41.6, 41.0, 37.4, 37.0, 36.4, 35.5, 32.3, 32.0, 30.4, 28.1, 26.6, 26.5, 26.1, 26.0, 23.1, 23.3, 21.6, 18.4, 17.3, 16.9. ESI-MS (*m/z*): (M−H)^−^ = 819.40. Anal. Calcd. for C_51_H_64_O_9_: C, 74.61; H, 7.86; O, 17.54%. Found: C, 74.68; H, 17.60; O, 17.59%.

#### 3.5.2. General Procedure for the Synthesis of Compounds **18c**–**d**

A stirred suspension of **17** (10 mmol) in acetyl chloride was heated up to 50 °C for 1 h resulting in a clear solution. Excess acetyl chloride was removed under vacuum and the residue triturated using diethyl ether. The residue was filtered and dried *in vacuo* to get glycyrrhetinic acid chloride. To a stirred, ice-cooled, solution of glycyrrhetinic acid chloride (20 mL, 10 mmol), a solution of CCM or BCM (20 mmol) in dry CHCl_3_ (30 mL) was added dropwise, and the resulting solution was stirred for 10 min at 0 °C. Then, TEA (10 mmol) was added to the solution and stirred at room temperature for 10 h. After completion of the reaction, as indicated by TLC, the mixture was washed with sodium bicarbonate (1M) and then with brine. The organic layer was dried with anhydrous Na_2_SO_4_ and evaporated to dryness under vacuum. Purification by chromatography on silica (CHCl_3_:MeOH = 20:1) gave **18c**–**d** as a yellow solid.

*Curcumin-di-glycyrrhetinic acid acetate* (**18c**). Yield 65%. m.p. 238–239 °C. ^1^H-NMR (400 MHz, CDCl_3_) δ 7.66 (d, *J =* 15.8 Hz, 2H), 7.21–7.13 (m, 6H), 6.65 (d, *J =* 15.8 Hz, 2H), 5.80 (s, 2H), 4.55 (dd, *J =* 12.2, 5.6 Hz, 2H), 2.85 (d, *J =* 14.8 Hz, 2H), 2.38 (s, 2H), 2.25(s, 6H), 2.15 (d, *J =* 15.6 Hz, 2H), 2.08–1.67 (m, 24H), 1.52–1.10 (m, 14H), 1.33 (s, 6H), 1.28(s, 6H), 1.26 (s, 6H), 1.15 (s, 3H), 0.88 (s, 12H), 0.84 (s, 6H). ^13^C-NMR (CDCl_3_): δ 200.8, 198.9, 176.3, 171.6, 170.2, 151.7, 143.9, 141.3, 131.9, 130.6, 128.3, 125.4, 124.7, 121.9, 121.9, 110.9, 80.6, 61.3, 55.8, 54.8, 51.9, 48.2, 44.6, 43.6, 41.4, 41.1, 37.6, 37.1, 36.3, 35.8, 32.5, 32.1, 30.5, 28.5, 26.9, 26.7, 26.4, 25.7, 24.2, 23.7, 21.3, 18.7, 17.3, 16.8. ESI-MS (*m/z*): (M−H)^−^ = 1,355.80. Anal. Calcd. for C_85_H_112_O_14_: C, 75.19; H, 8.31; O, 16.50%. Found: C, 75.20; H, 8.37; O, 16.59%.

*Bisdesmethoxycurcumin-di-glycyrrhetinic acid acetate* (**18d**). Yield 62%. m.p. 245–246 °C. ^1^H-NMR (400 MHz, CDCl_3_): δ 7.69 (d, *J =* 16.4 Hz, 2H), 7.59 (d, *J =* 12.0 Hz, 4H), 7.18 (d, *J =*12.2 Hz, 4H), 6.63 (d, *J =* 15.8 Hz, 2H), 4.70 (dd, *J =* 16.2, 8.2 Hz, 2H), 2.86 (d, *J =* 14.8 Hz, 2H), 2.38 (s, 2H), 2.28(s, 6H), 2.17 (d, *J =* 15.6 Hz, 2H), 2.11–1.78 (m, 20H), 1.58–1.19 (m, 14H), 1.37 (s, 6H), 1.30 (s, 6H), 1.29 (s, 6H), 1.19 (s, 3H), 0.89 (s, 12H), 0.87 (s, 6H). ^13^C-NMR (CDCl_3_): δ 200.8, 198.9, 175.3, 170.6, 169.2, 168.7, 142.9, 140.3, 130.7, 130.2, 127.3, 124.4, 122.7, 121.9, 120.4, 111.5, 80.3, 60.2, 52.8, 50.9, 47.2, 44.2, 43.1, 41.0, 40.1, 36.6, 36.1, 35.3, 34.8, 32.5, 32.1, 30.5, 28.5, 26.5, 26.0, 25.7, 24.2, 23.4, 21.1, 18.2, 17.0, 16.8. ESI-MS (*m/z*): (M−H)^−^ = 1,355.80. Anal. Calcd. for C_83_H_108_O_12_: C, 76.82; H, 8.39; O, 14.79%. Found: C, 76.80; H, 8.30; O, 14.69%.

#### 3.5.3. General Procedure for the Synthesis of Compounds **19a**–**d**

A solution of **19a**–**d** (1 mmol) and CH_3_ONa (10 mmol) in MeOH (10 mL) was stirred at room temperature overnight. The solvent was evaporated and the residue diluted with water and HCl (1 M) and extracted with CHCl_3_. The organic layers were dried, and the solvent was evaporated *in vacuo*. The residue was purified by flash chromatography on silica (CHCl_3_:MeOH = 20:1) to give **19a**–**d** as a yellow powder.

*Curcumin-mono-glycyrrhetinic acid* (**19a**). Yield 62%. 198–199 °C. ^1^H-NMR (400 MHz, CDCl_3_): δ 7.69 (d, *J =* 8.0 Hz, 1H), 7.57 (d, *J =* 8.0 Hz, 1H), 7.45–7.28 (m, 4H), 7.17 (d, *J =* 8.0 Hz, 1H), 7.04 (d, *J =* 16.0 Hz, 1H), 6.89 (d, *J =* 8.4 Hz, 1H), 6.84 (d, *J =* 16.2 Hz, 1H), 5.76 (s, 1H), 4.59 (dd, *J =* 12.8, 5.4 Hz, 1H), 3.87 (s, 3H), 3.84 (s, 3H), 2.85 (d, *J =* 14.0 Hz, 1H), 2.38 (s, 2H), 2.19 (d, *J =* 14.0 Hz, 1H), 2.09(s, 3H), 2.15–1.76 (m, 12H), 1.47–1.15 (m, 7H), 1.38 (s, 3H), 1.28(s, 3H), 1.25(s, 3H), 1.17 (s, 3H), 0.88 (s, 6H), 0.84 (s, 3H). ESI-MS (*m/z*): (M−H)^−^ = 819.40. Anal. Calcd. for C_51_H_64_O_9_: C, 76.82; H, 8.39; O, 14.79%. ^13^C-NMR (CDCl_3_): δ 200.8, 198.9, 176.3, 170.6, 151.5, 149.1, 147.9, 142.8, 141.1, 131.8, 130.4, 128.3, 127.6, 124.4, 122.9, 121.9, 116.8, 111.9, 110.9, 78.6, 61.3, 56.1, 55.8, 54.6, 51.9, 48.2, 44.6, 43.6, 41.4, 41.1, 38.9, 36.7, 35.6, 33.2, 32.5, 30.6, 28.5, 27.4, 26.9, 26.5, 26.0, 25.4, 23.2, 18.8, 17.6, 17.0. Found: C, 76.80; H, 8.30; O, 14.69%. Found: C, 76.80; H, 8.31; O, 14.71%.

*Bisdesmethoxycurcumin-mono-glycyrrhetinic acid* (**19b**). Yield 58%. 208–209 °C. ^1^H-NMR (400 MHz, CDCl_3_): δ 7.88 (d, *J =* 8.5 Hz, 2H), 7.63 (d, *J =* 8.6 Hz, 2H), 7.39 (d, *J =* 8.5 Hz, 2H), 6.99 (d, *J =* 16.0 Hz, 1H), 6.89 (d, *J =* 8.6 Hz, 2H), 6.78 (d, *J =* 16.2Hz, 1H), 2.88 (d, *J =* 14.0 Hz, 1H), 2.38 (s, 1H), 2.19 (d, *J =* 13.9 Hz, 1H), 2.11(s, 3H), 2.11–1.69 (m, 12H), 1.40–1.19 (m, 7H), 1.39 (s, 3H), 1.28 (s, 3H), 1.24 (s, 3H), 1.17 (s, 3H), 0.87 (s, 6H), 0.85 (s, 3H). ^13^C-NMR (CDCl_3_): δ 201.2, 198.8, 177.3, 171.6, 152.5, 149.3, 147.6, 142.4, 141.3, 131.5, 130.6, 128.5, 126.6, 125.2, 121.9, 121.2, 117.8, 111.9, 110.5, 78.6, 61.3, 55.8, 54.6, 51.9, 48.2, 44.6, 43.6, 42.4, 41.5, 38.5, 36.5, 35.6, 33.6, 32.2, 30.3, 28.5, 27.9, 26.8, 26.4, 26.2, 25.5, 23.3, 18.9, 17.9, 17.4. ESI-MS (*m/z*): (M−H)^−^ = 759.40. Anal. Calcd for: C, 77.34; H, 7.95; O, 14.72%. Found: C, 77.40; H, 8.01; O, 14.68%.

*Curcumin-di-glycyrrhetinic acid* (**19c**). Yield 66%. 217–219 °C. ^1^H-NMR (400 MHz, CDCl_3_): δ 7.84 (d, *J =* 8.7 Hz, 2H), 7.59 (d, *J =* 8.6 Hz, 2H), 7.38 (d, *J =* 8.7 Hz, 2H), 6.98 (d, *J =* 16.2 Hz, 1H), 6.89 (d, *J =* 8.5 Hz, 2H), 6.78 (d, *J =* 16.0Hz, 1H), 3.90 (s, 6H), 2.89 (d, *J =* 16.0 Hz, 2H), 2.38 (s, 2H), 2.20 (d, *J =* 16.0 Hz, 2H), 2.07 (s, 3H), 2.09–1.57 (m, 24H), 1.55–1.19 (m, 7H), 1.35 (s, 6H), 1.28 (s, 6H), 1.24 (s, 6H), 1.19 (s, 6H), 0.89 (s, 12H), 0.86 (s, 6H). ^13^C-NMR (CDCl_3_): δ 200.8, 198.5, 177.3, 171.6, 153.5, 150.1, 148.9, 143.8, 141.9, 132.2, 131.4, 129.3, 128.2, 125.4, 123.6, 122.3, 117.8, 113.9, 111.5, 79.5, 61.8, 57.2, 56.9, 55.6, 52.9, 49.2, 45.6, 44.1, 42.0, 41.2, 38.9, 36.9, 35.6, 33.2, 31.5, 30.6, 28.8, 27.3, 26.9, 26.5, 26.2, 25.8, 23.2, 19.8, 18.6, 17.6. ESI-MS (*m/z*): (M−H)^−^ = 1,243.70. Anal. Calcd. for C_79_H_104_O_12_: C, 76.17; H, 8.42; O, 15.41%. Found: C, 76.10; H, 8.47; O, 15.46%.

*Bisdesmethoxycurcumin-di-glycyrrhetinic acid* (**19d**). Yield 66%. 225–226 °C. ^1^H-NMR (400 MHz, CDCl_3_): δ 7.84 (d, *J =* 8.6 Hz, 2H), 7.59 (d, *J =* 8.5 Hz, 2H), 7.33 (d, *J =* 8.6 Hz, 2H), 6.95 (d, *J =* 16.4 Hz, 1H), 6.85 (d, *J =* 8.5 Hz, 2H), 6.79 (d, *J =* 16.2 Hz, 1H), 2.88 (d, *J =* 13.9 Hz, 2H), 2.47 (s, 2H), 2.17 (d, *J =* 14.0 Hz, 2H), 2.14–1.77 (m, 24H), 1.55–1.14 (m, 14H), 1.37 (s, 6H), 1.28(s, 6H), 1.24(s, 6H), 1.18 (s, 6H), 0.88 (s, 12H), 0.85 (s, 6H). ^13^C-NMR (CDCl_3_): δ 202.2, 199.2, 179.3, 172.6, 153.6, 149.6, 147.6, 142.4, 141.3, 131.5, 130.6, 128.5, 126.6, 125.2, 121.9, 121.2, 117.8, 111.9, 110.5, 78.6, 61.3, 56.3, 54.8, 51.6, 48.5, 44.2, 43.8, 42.6, 41.6, 38.8, 36.2, 35.2, 33.2, 32.0, 30.5, 28.2, 27.9, 26.5, 26.0, 25.9, 25.5, 23.6, 18.5, 17.4, 17.1. ESI-MS (*m/z*): (M−H)^−^ = 1,211.75. Anal. Calcd. for C_79_H_104_O_10_: C, 78.18; H, 8.64; O, 13.18%. Found: C, 78.10; H, 8.67; O, 13.12%.

### 3.6. Assay for DPPH-Scavenging Activity

Antioxidant activity was determined as radical-scavenging ability using the stable radical, DPPH. Briefly, 100 µL of ethanol solutions of the synthesized curcumin or bisdesmethoxycurcumin derivatives (5 µM, 10 µM, 20 µM, 40 µM, 80 µM and 160 µM) were added to 100 µL of an ethanol solution of DPPH radicals (100 µM). After 30 min of incubation at 25 °C in a dark environment, the absorbance was recorded at 517 nm, using ethanol as a blank for baseline correction. CCM and BCM were used as references, and their concentrations were maintained to that of the synthesized compounds. Sample concentrations providing 50% inhibition (IC_50_) were calculated from the graph of inhibition percentage plotted against concentration. These experiments were carried out in triplicate, and the resulting values were averaged. The radical-scavenging activity (%) was calculated using the following formula:

Radical-scavenging activity (%) = [(Ac − As)/Ac] × 100]
(1)

where Ac is the absorbance of the control and As is the absorbance of the tested sample after 30 min.

### 3.7. Antibacterial Assay

The *in vitro* antibacterial activity of each curcuminoid bioconjugate against Gram-positive cocci (*S. aureus* and *S. viridans*) and Gram-negative bacilli (*E. coli* and *E. cloacae*) was evaluated by the microdilution broth susceptibility test method. The stock solutions of the conjugates along with curcuminoids were prepared in DMSO. The tubes bore different concentrations (1 µg/mL, 1.25 µg/mL, 2 µg/mL, 2.5 µg/mL, 5 µg/mL and 10 µg/mL). All of the compounds and control tubes were incubated at 37 °C for 24 h. After incubation, the antibacterial activity of molecules in the tube was detected by the lack of turbidity, which indicated the inhibition of bacterial growth. The concentration in the tube with the highest dilution showing no turbidity has been reported as the MIC. Each test was performed in triplicate, and the MIC reported represents the result of at least three repetitions.

### 3.8. Anticancer Assays

Hep-G2, LX-2, SMMC-7221 and MDA-MB-231 cells were seeded at a density of 2 × 10^3^ cells/well. These cells were incubated at 37 °C under a humidified atmosphere containing 5% CO_2_ for 24 h before treatment with compounds. The cells were treated with various concentrations (1 µM, 5 µM, 10 µM and 20 µM) of the curcumin and bisdesmethoxycurcumin derivatives for 72 h. After treatment, media was removed, and the cells were washed with 3-[4,5-dimethylthiazol-2-yl]-2,5-diphenyl tetrazolium bromide (MTT). Approximately 20 µL of the MTT solution were added to each well followed by incubation for 6 h at 37 °C. The media was removed, followed by the addition of 200 µL of DMSO. The absorbance was recorded on a microplate reader at 540 nm. The cell viability (%) was calculated as the ratio of the number of surviving cells upon treatment with the test compounds compared to the blank.

## 4. Conclusions

Three series of curcuminoid derivatives were synthesized through conjugation of bioactive compounds via ester bonds. The radical-scavenging assay by the DPPH method clearly indicated that all curcuminoid derivatives have lower IC_50_ values than the curcuminoids. The results of the bioassay showed that compounds containing one selenomethionine molecule (**11b**) attached on the hydroxy group of curcuminoids showed the highest antimicrobial activity. As the starting materials showed broad anti-tumor activities, we chose four different human cancer cell lines to evaluate the curcuminoid derivatives. Three series of curcuminoid derivatives all inhibited the proliferation of all cancer cells. The results suggest that the attempt to apply a structure combination to discover more efficient, less toxic and more effective antibacterial, antioxidant and antiproliferative lead compounds is viable.
